# P-587. Data for Action: Using Medicare Part D Public Use Files for Tracking & Reporting Antibiotic Use at the National, State & Local Levels

**DOI:** 10.1093/ofid/ofaf695.801

**Published:** 2026-01-11

**Authors:** Sarah Kabbani, Christine Kim, Rachel Schaefer, Anna Garcia, Jeanette Prignano, Nicole A Mintz, Garret Hino, Dhivya Selvaraj, Marcia Glick, Rekha Murthy, Simisola Teye, Madeline Powers, Susan Klammer, Corey Lance, Kranthi Swaroop Koonisetty, Qaiser Jahan, Ryan Briola, Teah Snyder, Katarina Kamenar, Erica Stohs, Destani J Bizune, Gouin Katryna

**Affiliations:** Centers for Disease Control and Prevention, Atlanta, GA; CDC Division of Healthcare Quality Promotion, Atlanta, Georgia; Colorado Department of Public Health and Environment, Denver, Colorado; Healthcare Associated Infections and Antimicrobial Resistance Program, Connecticut Department of Public Health, Hartford, Connecticut; Healthcare Associated Infections and Antimicrobial Resistance Program, Connecticut Department of Public Health, Hartford, Connecticut; Hawaii State Department of Health (HDOH), Disease Outbreak Control Division, Honolulu, Hawaii; Hawaii State Department of Health (HDOH), Disease Outbreak Control Division, Honolulu, Hawaii; Infectious Disease Epidemiology & Prevention Division, Indiana Department of Health, Indianapolis, Indiana; Antibiotic Stewardship Program, Healthcare Outreach Unit, Acute Communicable Diseases Control, Los Angeles County Department of Public Health, Los Angeles, California; Antibiotic Stewardship Program, Healthcare Outreach Unit, Acute Communicable Diseases Control, Los Angeles County Department of Public Health, Los Angeles, California; Antibiotic Stewardship Program, Healthcare Outreach Unit, Acute Communicable Diseases Control, Los Angeles County Department of Public Health, Los Angeles, California; Minnesota Department of Health, Saint Paul, Minnesota; Minnesota Department of Health, Saint Paul, Minnesota; Healthcare-Associated Infections/Antimicrobial Resistance Program, Missouri Department of Health and Senior Services, Jefferson City, Missouri; Division of Healthcare Associated Infection Prevention, Pennsylvania Department of Health, Harrisburg, Pennsylvania; Division of Healthcare Associated Infection Prevention, Pennsylvania Department of Health, Harrisburg, Pennsylvania; Division of Healthcare Associated Infection Prevention, Pennsylvania Department of Health, Harrisburg, Pennsylvania; Division of Healthcare Associated Infection Prevention, Pennsylvania Department of Health, Harrisburg, Pennsylvania; Washington State Department of Health, Shoreline, Washington; Creighton University Medical Center, Omaha, NE; Eagle Global Scientific/CDC, Atlanta, GA; CDC Division of Healthcare Quality Promotion, Atlanta, Georgia

## Abstract

**Background:**

Public health antibiotic stewardship programs utilize different data sources to support antibiotic use (AU) surveillance and inform stewardship priorities. The Centers for Medicare and Medicaid Services (CMS) Medicare Part D Prescribers Public Use Files (PUFs) contain annual summaries of prescription drug claims by state and provider for all drugs, antibiotics, opioids, and antipsychotics. In 2022, the Centers for Disease Control and Prevention (CDC) initiated a community of practice on using PUFs for AU surveillance. We describe national and local use cases of PUFs to assess and inform public health stewardship activities.

**Figure 1:**
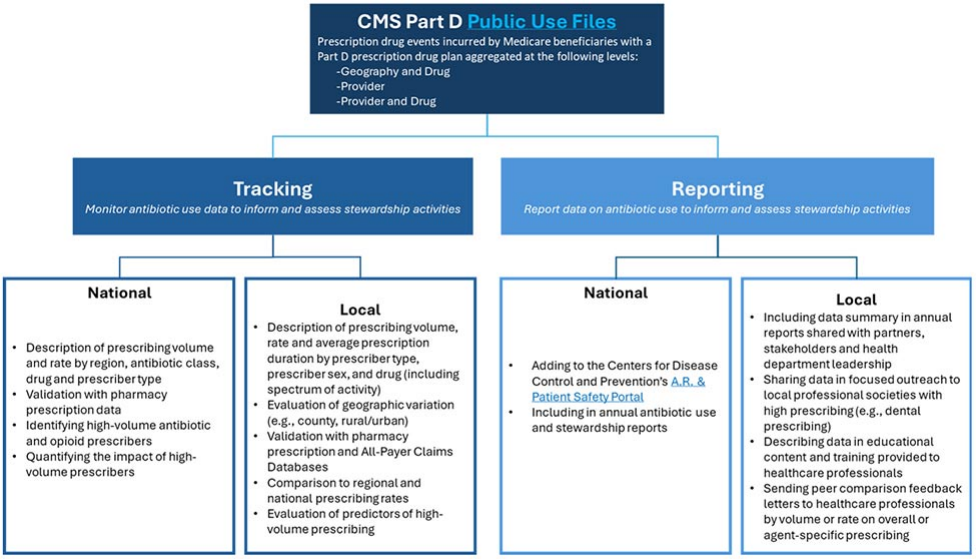
Summary of Tracking and Reporting Centers for Medicare and Medicaid Services (CMS) Part D Public Use Files National and Local Public Health Antibiotic Stewardship Use Cases

**Figure 2:**
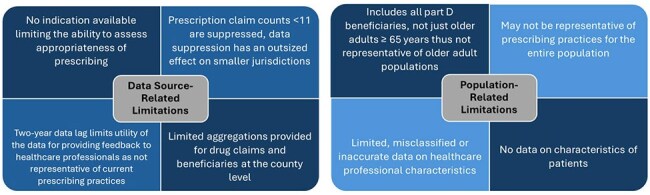
Data Source and Population Related Limitations of Centers for Medicare and Medicaid Services Part D Public Use Files

**Methods:**

The CMS PUFs community of practice, comprised of 13 jurisdictions (11 state and 2 local health departments), met three times in 2024. CDC held nine one-on-one technical calls with jurisdictions. We summarized use cases and data limitations from analyses of CMS PUFs. We categorized national and local use cases under the Tracking and Reporting core elements as defined in the Core Elements of Antibiotic Stewardship for Health Departments guidance (released in 2023).

**Results:**

CMS PUFs have been used for tracking antibiotic prescribing practices by provider type, antibiotic, and geography at national and local levels. Specifically, these data have been used to identify counties with high prescribing volume and evaluate predictors of high prescribing. AU rates were compared across region, state, and urban versus rural areas and validated with other data. Data have been shared publicly and internally with health department leadership, partners, and stakeholders, and used to inform education and peer-comparison feedback letters to prescribers. (Figure 1) Limitations associated with using PUFs for engaging prescribers included the lack of prescription indication, misclassification of practice location, data timeliness and representativeness. (Figure 2)

**Conclusion:**

CMS PUFs are no-cost data that can be leveraged at a national and local level to conduct AU surveillance for Medicare beneficiaries and support feedback interventions to improve prescribing. Minimizing time lag and providing county-level aggregations will help improve the utility of these data for public health antibiotic stewardship.

**Disclosures:**

Christine Kim, PhD, Moderna, Inc.: Epidemiologist|Moderna, Inc.: Stocks/Bonds (Public Company) Erica Stohs, MD MPH, bioMerieux, Inc.: Grant/Research Support|Merck Co, Inc: Grant/Research Support

